# Predicting value of ABCD2 in early ischemic stroke in patients diagnosed with transient ischemic attack

**Published:** 2012-11

**Authors:** Mojtaba Chardoli, Alireza Khajavi, Mohsen Nouri, Vafa Rahimi-Movaghar

**Affiliations:** ^*a*^Deparment of Neurosurgery - Sina Hospital, Tehran University of Medical Sciences, Tehran, Iran.; ^*b*^Research Centre for Neural Repair, University of Tehran, Tehran, Iran.

## Abstract

**Background:**

As a significant number of patients diagnosed with transient ischemic attack (TIA) at emergency department are at risk to develop TIA or cerebral vascular accident (CVA), several attempts have been made to figure out a predictive method to detect those at higher risk of such attacks. Therefore, the present study was aimed to evaluate the role of ABCD2 scoring including age, blood pressure, clinical features, duration, and diabetes mellitus (DM), in predicting short term outcome of the patients presenting with TIA.

**Methods:**

One hundred consecutive patients who have attended Hazrat Rasoul Akram Hospital (Kermanshah, Iran) during 2009 to 2010 and diagnosed with TIA were enrolled in the study. Their ABCD2 scores were recorded. The incidences of death, CVA, and TIA during the first week after the attack were recorded.

**Results:**

Eleven patients suffered from new TIA/CVA after 1 week. Sensitivity and specificity of ABCD2 score for predicting CVA/TIA at cut-off point of the 4th day were 72.7% and 52.8%, respectively. At the same cut-off point for ABCD2, positive and negative predictive values were 16% and 94 %, respectively.

**Conclusions:**

Our results show that although patients with ABCD2 score greater than 4 were more likely to develop recurrent TIA/CVA in short term, those with lower scores are still susceptible to a considerable risk of TIA/CVA. Though ABCD2 as an easily applicable tool is very helpful in management of TIA patients at emergency department, but it should not be the only measure to rely on in our decision making.

**Keywords:**

Ischemic stroke, Transient ischemic attack, Cerebral vascular accident, ABCD2 scoring


					Volume 4, Suppl. 1 Nov 2012
Publisher: Kermanshah University of Medical Sciences
URL: http://www.jivresearch.org
Copyright:
Congress of Iranian Neurosurgeons, 14-16 November 2012, Kermanshah, Iran
Paper No. 60
Predicting value of ABCD2 in early ischemic stroke in
patients diagnosed with transient ischemic attack
Mojtaba Chardoli a
, Alireza Khajavi a
, Mohsen Nouri a,*
, Vafa Rahimi-Movaghar a,b
a
Deparment of Neurosurgery - Sina Hospital, Tehran University of Medical Sciences, Tehran, Iran.
b
Research Centre for Neural Repair, University of Tehran, Tehran, Iran.
Abstract:
Background: As a significant number of patients diagnosed with transient ischemic attack (TIA) at emergency
department are at risk to develop TIA or cerebral vascular accident (CVA), several attempts have been made to
figure out a predictive method to detect those at higher risk of such attacks. Therefore, the present study was aimed
to evaluate the role of ABCD2 scoring including age, blood pressure, clinical features, duration, and diabetes mellitus
(DM), in predicting short term outcome of the patients presenting with TIA.
Methods: One hundred consecutive patients who have attended Hazrat Rasoul Akram Hospital (Kermanshah, Iran)
during 2009 to 2010 and diagnosed with TIA were enrolled in the study. Their ABCD2 scores were recorded. The
incidences of death, CVA, and TIA during the first week after the attack were recorded.
Results: Eleven patients suffered from new TIA/CVA after 1 week. Sensitivity and specificity of ABCD2 score for
predicting CVA/TIA at cut-off point of the 4th day were 72.7% and 52.8%, respectively. At the same cut-off point
for ABCD2, positive and negative predictive values were 16% and 94 %, respectively.
Conclusions: Our results show that although patients with ABCD2 score greater than 4 were more likely to develop
recurrent TIA/CVA in short term, those with lower scores are still susceptible to a considerable risk of TIA/CVA.
Though ABCD2 as an easily applicable tool is very helpful in management of TIA patients at emergency department,
but it should not be the only measure to rely on in our decision making.
Keywords:
Ischemic stroke, Transient ischemic attack, Cerebral vascular accident, ABCD2 scoring
* Corresponding Author at:
Mohsen Nouri: Deparment of Neurosurgery, Sina Hospital, Tehran University of Medical Sciences, Tehran, Iran. Tel: 09375325455,
Email: m_noori@razi.tums.ac.ir, (Nouri M.).

				

